# Composite of Long Polymer Chains and Structured Silica Particles Achieves High Modulus and Low Hysteresis

**DOI:** 10.1021/acsmaterialsau.5c00258

**Published:** 2026-04-02

**Authors:** Matthew Wei Ming Tan, Chenghai Li, Masako Yoshihara Yang, Mikaya Cooper Parente, Yakov Kutsovsky, Zhigang Suo

**Affiliations:** † John A. Paulson School of Engineering and Applied Sciences, 1812 Harvard University, Cambridge, Massachusetts 02138, United States; ‡ School of Materials Science and Engineering, 541848 Nanyang Technological University, 50 Nanyang Avenue, Singapore 639798, Singapore; § Expert-in-Residence, Office of Technology Development, Harvard University, Cambridge, Massachusetts 02138, United States

**Keywords:** natural rubber, composites, silica particles, modulus, hysteresis

## Abstract

Many applications require rubber-like materials to fulfill two important requirements: high modulus to resist excessive deformation and low hysteresis to reduce energy loss. High modulus is commonly realized by mixing rubber with inorganic fillers, but the fillers also increase the hysteresis. This conflict between the modulus and hysteresis presents a challenge in material development. Here, we show that a high modulus and low hysteresis are achieved simultaneously in a composite of long rubber chains and structured fillers. We prepare a model system using natural rubber latex with long polymer chains and structured fillers in which primary silica particles fuse into aggregates. Each aggregate has an open structure that occludes the rubber. The composite forms two networks: a network of cross-linked natural rubber chains and a network of percolated silica aggregates. The two networks intertwine with each other and interlink through covalent bonds. We measure the modulus and hysteresis of the composites prepared with various volume fractions of aggregates and molar fractions of the initiator. We discuss the mechanisms by which long rubber chains and structured silica aggregates lower hysteresis while maintaining a high modulus. This work provides insights into the development of rubber products for energy efficiency.

## Graphical abstract

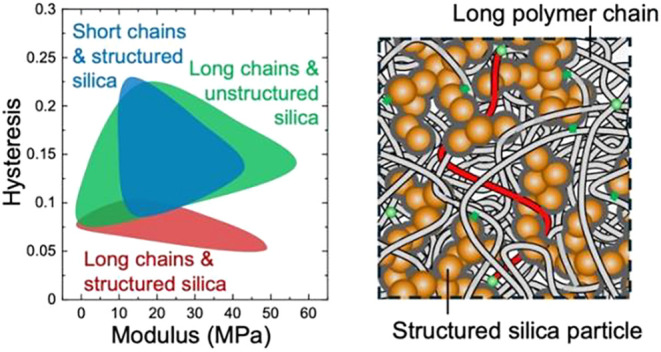

## 1 Introduction

In applications such as tires, gaskets, and soft robotics, rubber-like materials need to fulfill two important requirements: high modulus to resist excess deformation and low hysteresis to reduce energy loss.^1–4^ These two requirements often conflict. In particular, a potent method to amplify modulus is to mix rubber with inorganic fillers, such as carbon black and silica particles, but a filled rubber commonly has higher hysteresis than a neat rubber.^5,6^

In conventional manufacturing of rubber products, high-intensity mixing methods, such as two-roll milling and internal mixing, are typically used to mix fillers with rubber chains. However, these high-intensity processes inevitably shorten rubber chains.^7,8^ Consequently, reinforced rubbers often consist of a matrix of short chains. The synergy between the structure of fillers and long rubber chains on the modulus and hysteresis remains unexplored.

Here, we show that high modulus and low hysteresis can be achieved simultaneously in composites of long rubber chains and structured silica. We prepare composites of three types and compare them on the plane of modulus and hysteresis (Figure 1). For each type, samples are prepared with two variables: the molar fraction of the initiator, *C*, and the volume fraction of fillers, *F*. Modulus is determined by the slope of the stress–stretch curve at small stretches. Hysteresis is determined by the ratio of the loss modulus to storage modulus, *E*″/*E*′, using a dynamic mechanical analyzer (DMA) at 1 Hz, 1% strain, and room temperature. At a comparable modulus, the composite of long rubber chains and structured silica has lower hysteresis than the composite of short rubber chains and structured silica, as well as the composite of long chains and unstructured silica. For example, at a modulus of ∼40 MPa, the hysteresis is ∼0.06 for the composite with long chains and structured silica, ∼0.12 for the composite with short chains and structured silica, and ∼0.1 for the composite with long chains and unstructured silica. This twofold reduction in hysteresis may translate into appreciable energy savings.

**Figure 1:**
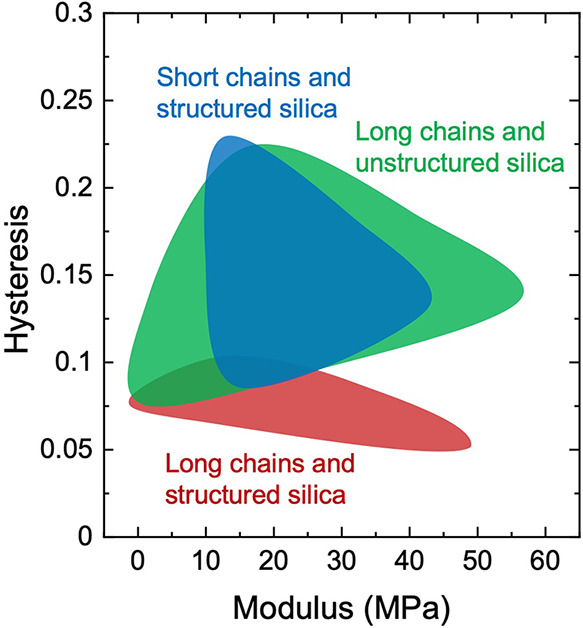
On the plane of modulus and hysteresis, three types of composites are compared: long-chain rubber filled with structured silica (red), short-chain rubber filled with structured silica (blue), and long-chain rubber filled with unstructured silica (green).

Polymer networks of long chains, sparse cross-links, and dense entanglements have recently been studied.^9–11^ The long chains reduce the fraction of dangling ends, the sparse cross-links amplify crack resistance, and the dense entanglements maintain modulus. When such a polymer network is filled with inorganic particles, both modulus and crack resistance are further amplified.^12–15^ In our previous works, rubbers were filled with unstructured particles, including silica and carbon black.^14,15^ In commercial products, however, rubbers are often filled with structured particles.^16^ Composites of long rubber chains and structured silica have not been studied before and are the focus of this paper. We find that long rubber chains and structured silica aggregates synergize to lower hysteresis and maintain a high modulus. We interpret this finding in terms of noncovalent interactions of three types: rubber–rubber, rubber–filler, and filler–filler.

## 2 Materials and Methods

### 2.1 Chemicals

Natural rubber latex (60 wt % rubber content) is purchased from Chemionics Corporation (OH). Dicumyl peroxide (DP, 32954) and toluene (244511) are purchased from Sigma-Aldrich. Unstructured silica particles functionalized with 3-(trimethoxysilyl)­propyl methacrylate are provided by Cabot Corporation. Structured silica particles (Aerosil R711) functionalized with methylacrylsilane and structured silica particles functionalized with octyltrimethoxysilane (Aerosil R805) are provided by Evonik. All materials are used as received.

### 2.2 Scanning Electron Microscopy

Scanning electron microscopy (SEM) images of the cross-section of a composite are taken using Zeiss Gemini 360 FE-SEM. To minimize charging, samples are coated with 10 nm thick Pt/Pd (80/20 composition) using a sputter coater.

### 2.3 Mechanical Tests

Stress–stretch curves are measured using a tensile tester (Instron 5966) at a stretch rate of 0.05 s^–1^. Samples are cut into a dumbbell shape by using a die cutter (Ace steel rule dies, ISO 527-2-5B). The stretch λ is defined as the deformed length divided by the undeformed length. The stress is defined as the applied force divided by the cross-sectional area of the undeformed sample. The Young’s modulus is defined as the slope of the stress–stretch curve at a small stretch (λ < 1.05). The rise of the stress–stretch curve is characterized by the stress at a stretch of λ = 2, i.e., 100% strain, termed the “100%-strain modulus.” ^17,18^

### 2.4 Dynamic Mechanical Analysis

Strain sweeps are measured by using a dynamic mechanical analyzer (DMA 1, Mettler Toledo). Strain sweeps are performed with amplitude of strain between 0.1 to 10%, at a frequency of 1 Hz and a temperature of 25 °C. The hysteresis is defined by the ratio of the loss modulus *E*″ to the storage modulus, *E*′, commonly written in terms of the phase shift δ: tan­(δ) = *E*″/*E*′.

To predict rolling resistance, strain sweeps are performed with a strain amplitude range between 0.1–10%, at a frequency of 10 Hz, and a temperature of 60 °C.

## 3 Processing of Composites

We choose natural rubber latex as the source for polymer chains owing to their high molecular weight of ∼1,000,000 g mol^–1^, low entanglement molecular weight of ∼3000 g mol^–1^, and low hysteresis.^19–22^ We chose silica aggregates functionalized with methacrylsilane, which enabled the silica aggregates to disperse in rubber and interlink with rubber by covalent bonds.

We compare silica particles of two types: unstructured and structured. In a powder of unstructured silica, individual silica particles form agglomerates through noncovalent interactions (Figure 2a). Upon mixing with rubber, the agglomerates break into individual particles, which disperse in the rubber matrix. The particles increase the viscosity of the mixture, which is desirable in applications such as coatings, adhesives, and sealants.^23,24^ In a powder of structured silica, primary silica particles are fused by covalent bonds into aggregates, which then assemble by noncovalent interactions into agglomerates (Figure 2b). Each aggregate has an open structure.^25^ Upon mixing with rubber, the agglomerates separate into aggregates, but the aggregates do not break; rubber fills the gaps between nearby aggregates and occludes the open structure within each aggregate.

**Figure 2:**
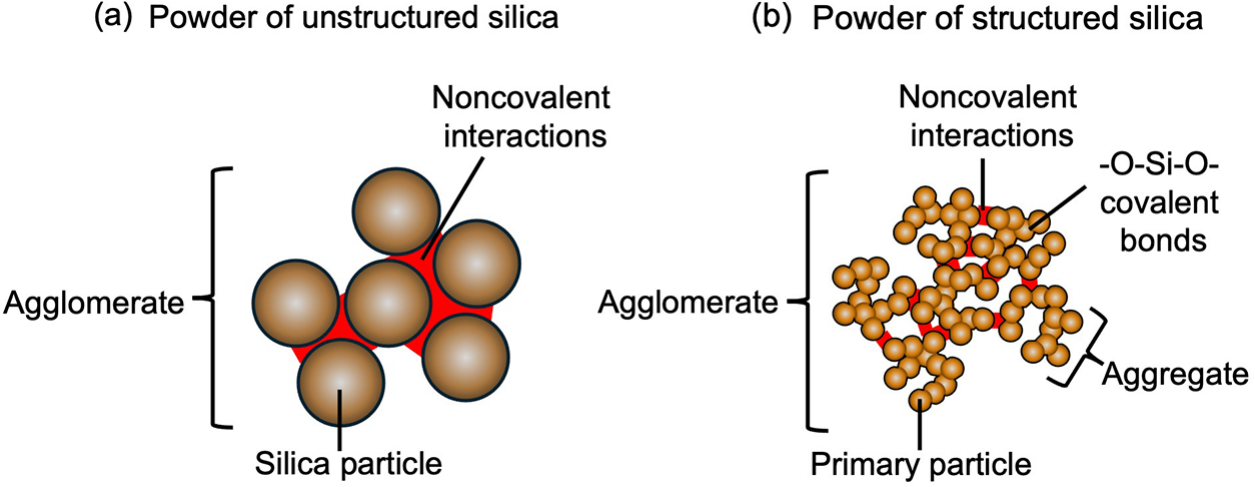
Powders of unstructured and structured silica. (a) In a powder of unstructured silica, silica particles form agglomerates by noncovalent interactions. (b) In a powder of structured silica, primary silica particles fuse into aggregates by covalent bonds, and the aggregates form agglomerates by noncovalent interactions. Each aggregate has an open structure.

To preserve long natural rubber chains and disperse silica aggregates, we prepared composites by liquid processing (Figure 3a). The thermal initiator, dicumyl peroxide (DP), is dissolved in toluene. Rubber latex is then mixed with the DP-toluene solution, with a latex-to-toluene mass ratio of 1:6, at 400 rpm and room temperature for 3 h. In the resulting DP-rubber-toluene solution, there remains a small amount of water surrounding the hydrophilic nonrubber components of natural rubber. Separately, a powder of structured silica is dispersed in toluene at a silica-to-toluene mass ratio of 1:21. The silica-toluene dispersion is then mixed with the DP-rubber-toluene solution at 400 rpm and room temperature for 30 min. During mixing, silica agglomerates break into aggregates. The resulting mixture is dried at room temperature for 4 days to form an uncured compound. The compound is cured by hot-pressing at 140 °C for 3 h. The silica aggregates are evenly distributed throughout the resulting composite and form a percolated network at *F* = 0.25 (Figure 3b,c).

**Figure 3:**
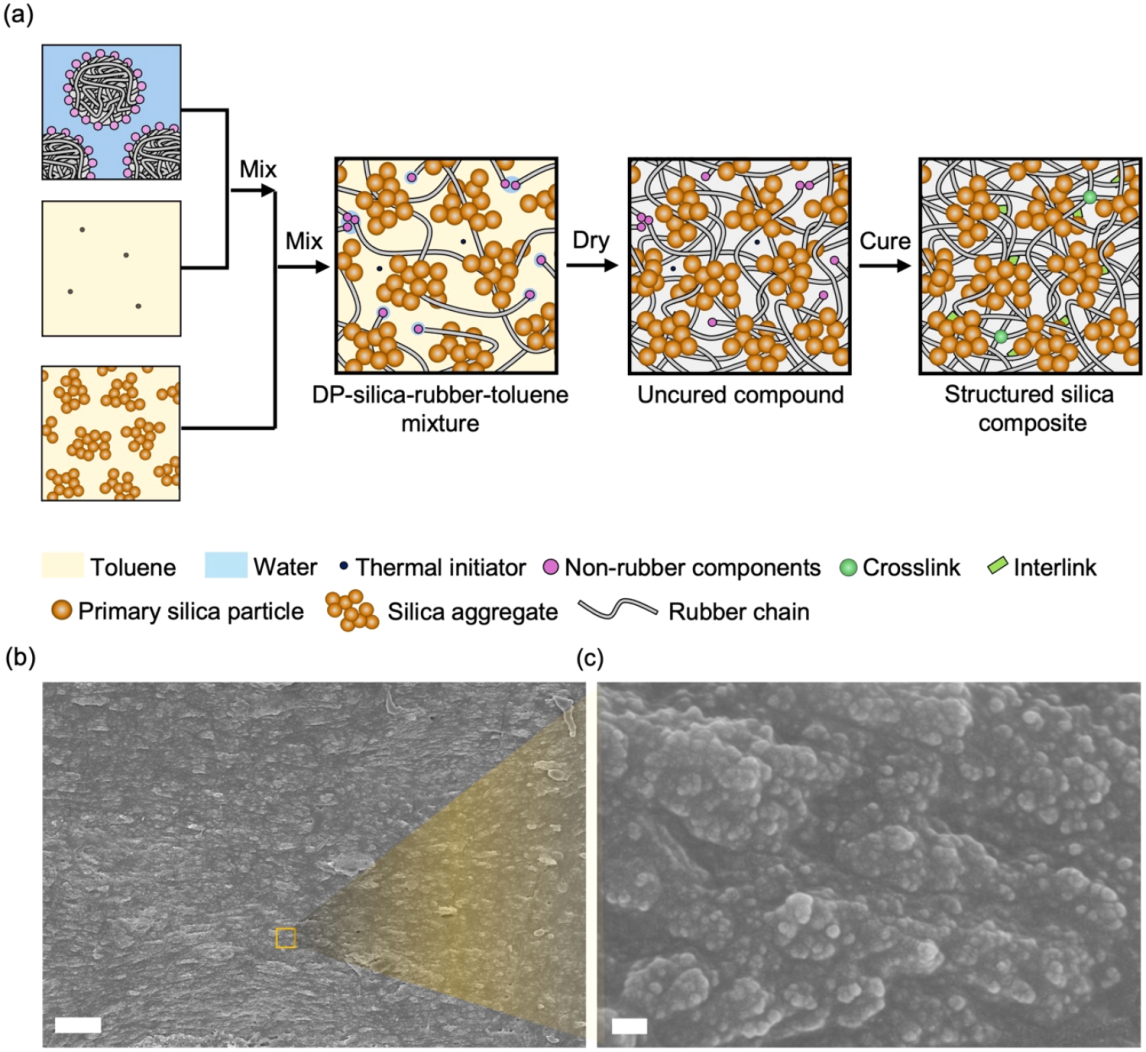
Processing composites of silica aggregates and long natural rubber chains. (a) Liquid processing. SEM image of the cross-section of structured silica composites of *C* = 1 × 10^–3^ and *F* = 0.25 with scale bars of (b) 5 μm and (c) 100 nm.

The initiator fraction *C* is defined by the molar ratio of DP to the repeat unit of the natural rubber chains. The volume fraction of aggregates in a composite is defined by *F* = (*m*_f_/ρ_f_)/((*m*_f_/ρ_f_)+(*m*_r_/ρ_r_)), where *m*_f_ is the mass of silica powder, ρ_f_ = 2.2 g cm^–3^ is the density of silica, *m*_r_ is the mass of dried rubber, and ρ_r_ = 0.95 g cm^–3^ is the density of rubber.

The composite assembles rubber chains and silica aggregates (Figure 4). The structured silica particles used in this work have a reported primary particle size of ∼12 nm and an aggregate size of ∼75–300 nm.^26,27^ Because silica aggregates are functionalized with methacrylsilane, after drying, the surface of each silica aggregate is covered by rubber. Rubber fills the thin gaps between nearby aggregates and occludes the open space within each aggregate. After curing, covalent bonds cross-link rubber chains to each other and interlink rubber chains to silica aggregates. Rubber chains adsorb onto the surface of silica aggregates to form a layer of bound rubber of a few nanometers in thickness.^28–30^ The bound rubber is stiff but is thin compared to the rubber between aggregates and occluded within aggregates. The long chains are sparsely cross-linked and densely entangled, forming a cross-linked network of rubber chains. Above a threshold volume fraction of *F* = 0.1, the silica aggregates percolate. In the percolated network, nearby silica aggregates are still separated by a thin layer of rubber. The composite consists of two networks: the network of cross-linked rubber chains and the network of percolated silica aggregates. The two networks intertwine and interlink.

**Figure 4:**
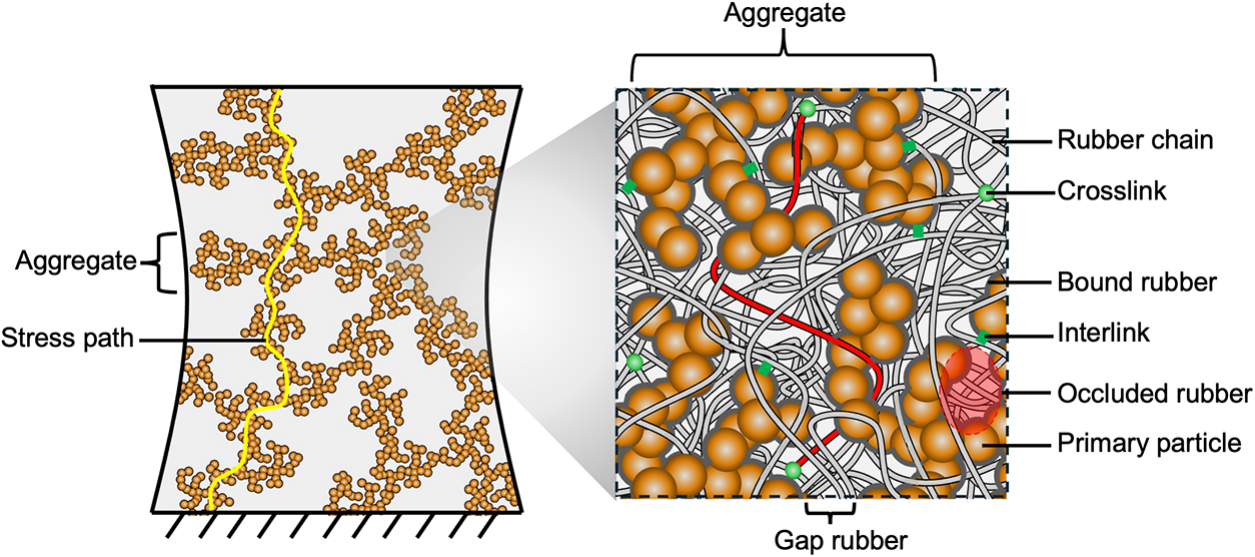
In a composite of long rubber chains and structured silica aggregates, the silica aggregates percolate into a network. The rubber chains separate nearby aggregates and occlude the open spaces within the aggregates.

We also prepare composites of long rubber chains and unstructured silica functionalized with acrylate groups.^15^ The powder of unstructured silica is easier to disperse in toluene than the powder of structured silica. Thus, we use a silica-to-toluene mass ratio of 1:7 to disperse unstructured silica compared to the ratio of 1:21 used to disperse structured silica. The two composites are prepared at the same initiator fraction, *C* = 1 × 10^–3^, but different volume fractions of silica particles, with *F* = 0.25 for structured silica, and *F* = 0.33 for unstructured silica.

We further studied the effects of chain length. Neat rubber or composites of the dried, uncured compound were prepared as before. The chains are cut short by passing the compound through a mill of two rollers with a gap of 1 mm.^11^ The milling is repeated 80 times at a rate of ∼10 rpm. After milling, the compound is cured by hot-pressing under the same conditions at 140 °C for 3 h.

In constructing Figure 1, we prepare long-chain rubber with structured silica of 1 × 10^–3^ ≤ *C* ≤ 1 × 10^–2^ and 0 ≤ *F* ≤ 0.25, short-chain rubber with structured silica of 1 × 10^–3^ ≤ *C* ≤ 1 × 10^–2^ and 0.15 ≤ *F* ≤ 0.25, and long-chain rubber with unstructured silica of 1 × 10^–3^ ≤ *C* ≤ 1 × 10^–2^ and 0 ≤ *F* ≤ 0.4.

## 4 Properties of Composites of Long Rubber Chains and Structured Silica Aggregates

After the composites of long rubber chains and structured silica aggregates are prepared, we measure the modulus and hysteresis of each sample and discuss the experimental data in terms of noncovalent interactions of three types: rubber–rubber, rubber–filler, and filler–filler.

### 4.1 Modulus

The stress–stretch curves are measured for composites with a fixed initiator fraction, *C* = 1 × 10^–3^, and various volume fractions of aggregates, *F* = 0, 0.1, 0.15, 0.2, and 0.25 (Figure 5a). The larger the volume fraction of aggregate *F*, the smaller the stretchability.

**Figure 5:**
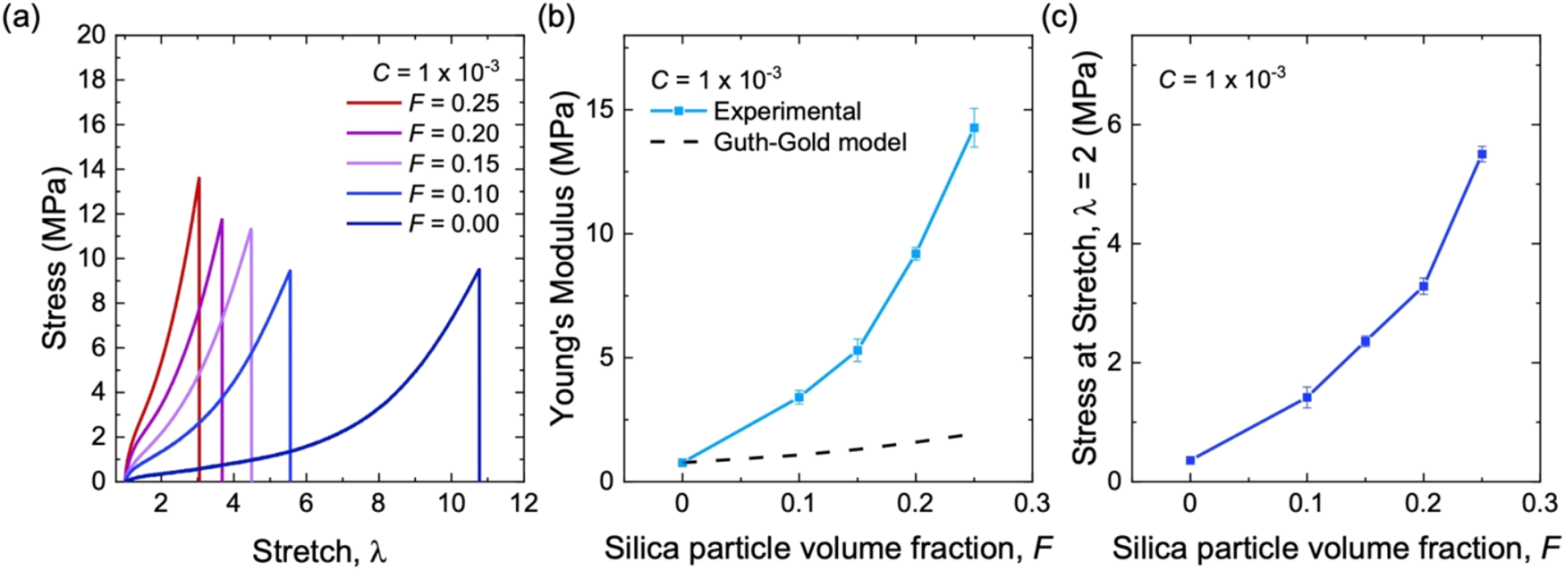
Stress–stretch behavior for composites of long-chain natural rubber and structured silica of various volume fractions, *F*. (a) Stress–stretch curves. (b) Young’s modulus as a function of volume fraction of aggregates. The dashed line represents the predictions by the Guth–Gold model. (c) Stress at a stretch of λ = 2 as a function of the volume fraction of aggregates. All samples have the same initiator fraction of *C* = 1 × 10^–3^.

As *F* increases, Young’s modulus increases (Figure 5b). The enhancement of Young’s modulus is evident at moderate volume fractions of aggregates (*F* < 0.15) and becomes pronounced when *F* further increases (*F* > 0.15). Young’s modulus rises from 0.77 MPa for neat natural rubber (*F* = 0) to 14 MPa for the composite at *F* = 0.25, corresponding to an 18-fold increase in stiffness.

Samples with higher *F* values show a steeper stress–stretch curve (Figure 5a). The 100%-strain modulus rises significantly with *F* (Figure 5c), and the dependence on *F* follows a trend similar to Young’s modulus.

Stress–stretch curves are also measured for composites with a fixed volume fraction of aggregates, *F* = 0.25, and various molar fractions of initiator, *C* = 1 × 10^–3^, 2 × 10^–3^, 3 × 10^–3^, 5 × 10^–3^, and 1 × 10^–2^ (Figure 6a). The stretchability decreases with increasing *C*. When *C* increases from 1 × 10^–3^ to 3 × 10^–3^, the modulus nearly plateaus around ∼15 MPa (Figure 6b). With the further increase of *C*, the modulus increases significantly, achieving ∼50 MPa at *C* = 1 × 10^–2^. The plateau of modulus results from the rubber matrix in which entanglements outnumber cross-links, and the modulus is set by entanglements.^14,15,31^ The 100%-strain modulus increases with *C*, but no plateau is observed over the range of *C* measured (Figure 6c). At a high initiator fraction of *C* = 1 × 10^–2^, the stretchability is reduced below λ = 2, and the 100%-strain modulus cannot be determined.

**Figure 6:**
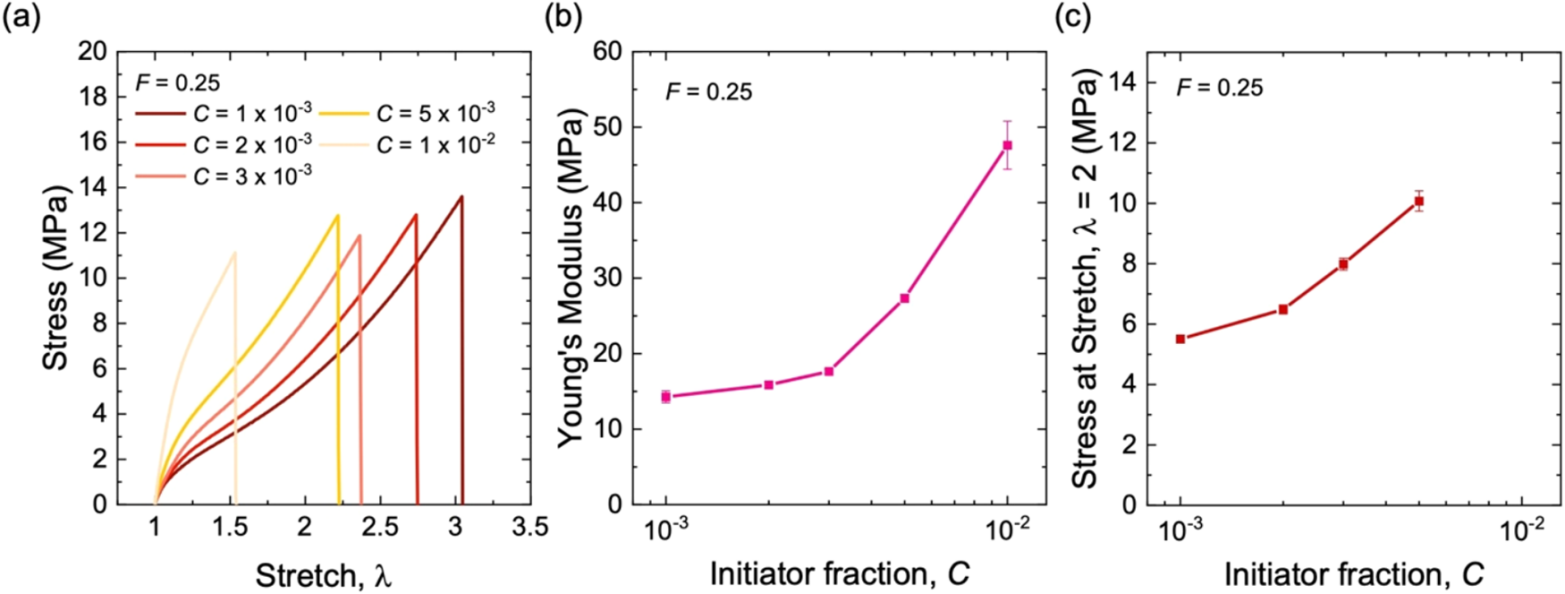
Stress–stretch behavior for a composite of long-chain natural rubber and structured silica of various initiator fractions, *C*. (a) Stress–stretch curves. (b) Young’s modulus. (c) Stress at stretch of λ = 2. All samples have the same volume fraction of aggregates at *F* = 0.25.

As noted above, when the volume fraction of silica aggregates exceeds the percolation threshold of *F* = 0.1, a composite forms two networks: a network of cross-linked rubber chains, and a network of percolated silica aggregates (Figure 4). The two networks intertwine with each other and interlink through covalent bonds. Consider a “unit cell” comprising two adjacent aggregates, the rubber gap between them, and the rubber occluded within their open internal voids. Because the rubber is incompressible and the aggregates are rigid, any contraction or extension of the gap rubber and the occluded rubber is constrained.^32,33^ The percolated network of silica aggregates is still deformable but is much stiffer than neat rubber.

Because the aggregates percolate, the modulus of the composite is significantly larger than that predicted by the Guth–Gold model, even at a low volume fraction of *F* = 0.1 (Figure 5b). This large increase in modulus indicates that aggregates form a percolated, stiff network. In the Guth–Gold model, Young’s modulus of a composite with rigid spherical particles in a linear elastic matrix is *E*(*C*, *F*) = *E*_m_(*C*)­(1 + 2.5*F* + 14.1*F* ^2^), where *E*(*C*, *F*) and *E*_m_(*C*) are Young’s moduli of the composite and the matrix, respectively, *C* is the cross-link density, and *F* is the volume fraction of particles. Our experimental data are consistent with previous reports that silica aggregates percolate at a threshold volume fraction of ∼0.1–0.15, depending on the fractal geometry.^34–36^

Nonetheless, if we model the composite by continuum mechanics, with rigid aggregates well-bonded in the neat rubber, the modulus of the composite scales with that of the neat rubber *E*(*C*, *F*) = α*E*_m_(*C*), where α is a dimensionless function of *F* and the arrangement of the fillers. In particular, for densely entangled and sparsely cross-linked long rubber chains, entanglements set the modulus of the neat rubber, leading to the plateau modulus.^31^ This plateau modulus of the matrix in turn leads to the plateau modulus of the composite (Figure 6b).

### 4.2 Hysteresis

Strain sweeps are performed with a strain amplitude range of 0.1–10%, and the hysteresis is defined by the ratio of the loss modulus *E*″ to the storage modulus, *E*′. This definition of hysteresis is applicable to the linear viscoelastic regime and has been widely adopted to characterize rubbers cycled under relatively small strains. Such small strains are comparable to the deformation in many applications, including tires.^37,38^

We compare composites with different volume fractions of silica aggregates *F* at the same initiator fraction, *C* = 1 × 10^–3^. The storage modulus *E*′ increases with *F* (Figure 7a). In particular, when *F* ≤ 0.15, *E*′ is nearly independent of the amplitude of strain. When *F* > 0.15, *E*′ decreases markedly with an increasing amplitude of strain. At a strain of 5%, *E*′ is comparable to Young’s modulus obtained from the slope of stress–stretch curves at λ < 1.05. Similarly, loss modulus *E*″ increases with *F* (Figure 7b). However, the strain dependence of *E*″ differs from that of *E*′ when *F* > 0.15, at which a peak in *E*″ emerges at intermediate strains.

**Figure 7:**
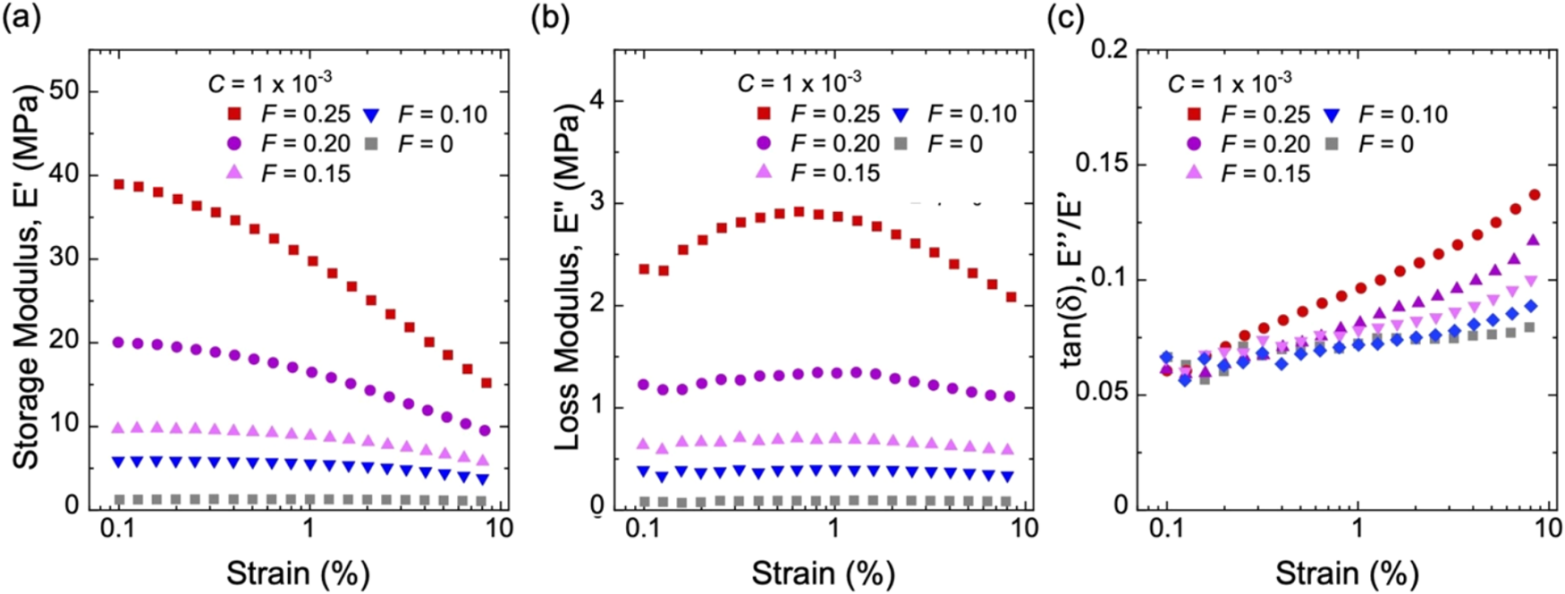
Dynamic mechanical properties of composites of long-chain natural rubber and structured silica of various volume fractions, *F*. (a) Storage modulus, *E*′. (b) Loss modulus, *E*″. (c) tan­(δ). All samples had the same initiator fraction of *C* = 1 × 10^–3^.

Because *E*′ and *E*″ are nearly strain-independent when *F* < 0.15, the hysteresis tan­(δ) increases mildly with increasing strain (Figure 7c). When *F* > 0.15, *E*′ decreases significantly with strain and *E*″ remains on the same order of magnitude, leading to a rise in hysteresis with strain. Notably, at small strains, e.g., 0.1–0.3%, the hysteresis is nearly independent of *F* and approaches that of the neat rubber (*F* = 0). At larger strains, e.g., 5∼10%, the hysteresis increases markedly with *F*, and is larger than that of the neat rubber.

We also test neat natural rubber (*F* = 0) and the composite (*F* = 0.25) at a high initiator fraction, *C* = 1 × 10^–2^. For neat rubber, *E*′ and *E*″, as well as tan­(δ), are independent of strain (Figure 8). The addition of silica aggregates amplifies both *E*′ and *E*″ across all strains (Figure 8a,b). At small strains, e.g., 0.1–0.3%, the difference in hysteresis of two materials is modest (Figure 8c). As strain increases, this difference becomes more pronounced.

**Figure 8:**
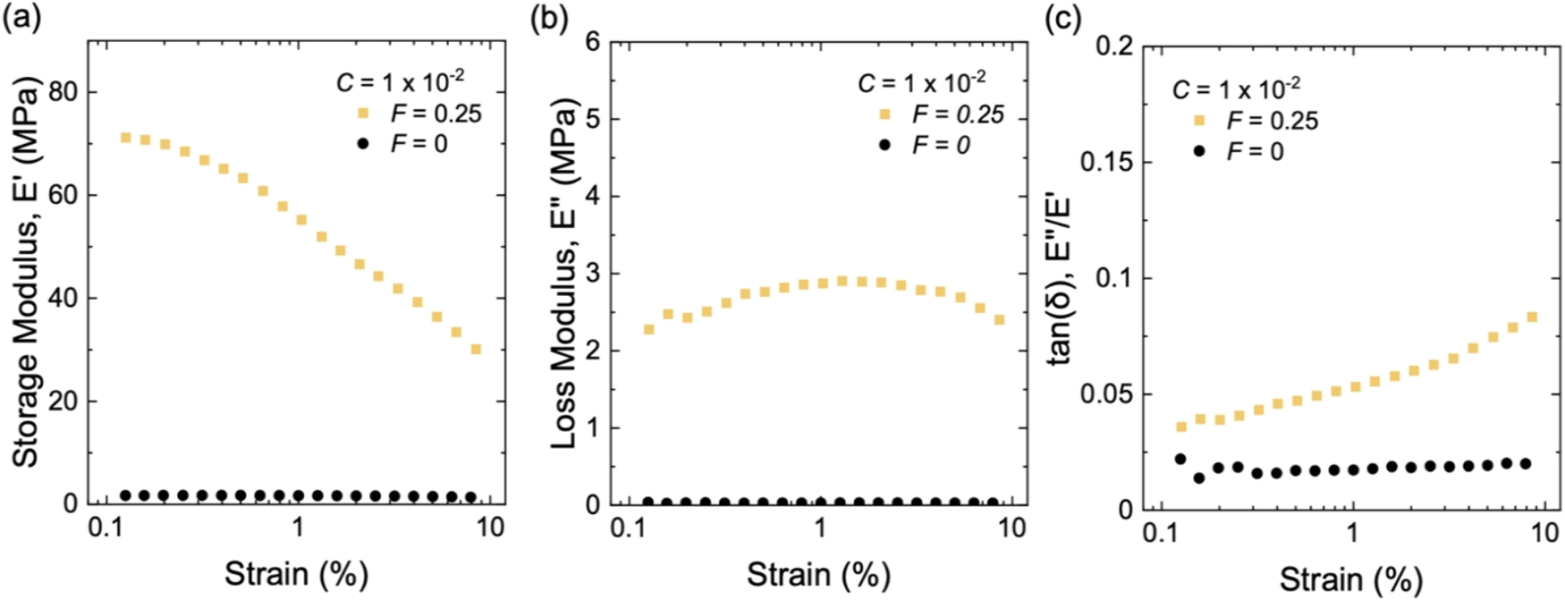
Dynamic mechanical properties for long-chain neat rubber (*F* = 0) and a composite of long-chain rubber and structured silica (*F* = 0.25) with the same initiator fraction of *C* = 1 × 10^–2^. (a) Storage modulus, *E*′. (b) Loss modulus, *E*″. (c) tan­(δ).

At a fixed volume fraction of aggregates, *F* = 0.25, we examine the effect of initiator fractions *C* on the hysteresis of the composites. For all values of *C*, *E*′ decreases with increasing strain (Figure 9a). On the other hand, over the entire range of measured strain, *E*′ increases with *C*. In particular, *E*′ increases mildly with *C* when *C ≤* 3 × 10^–3^, but increases significantly when *C* > 3 × 10^–3^. By contrast, *E*″ is nearly independent of *C* and peaks at an intermediate strain of ∼1% (Figure 9b). Consequently, the hysteresis, tan­(δ) = *E*″/*E*′, is dominated by the change in *E*′, so that tan­(δ) reduces with increasing *C* (Figure 9c).

**Figure 9:**
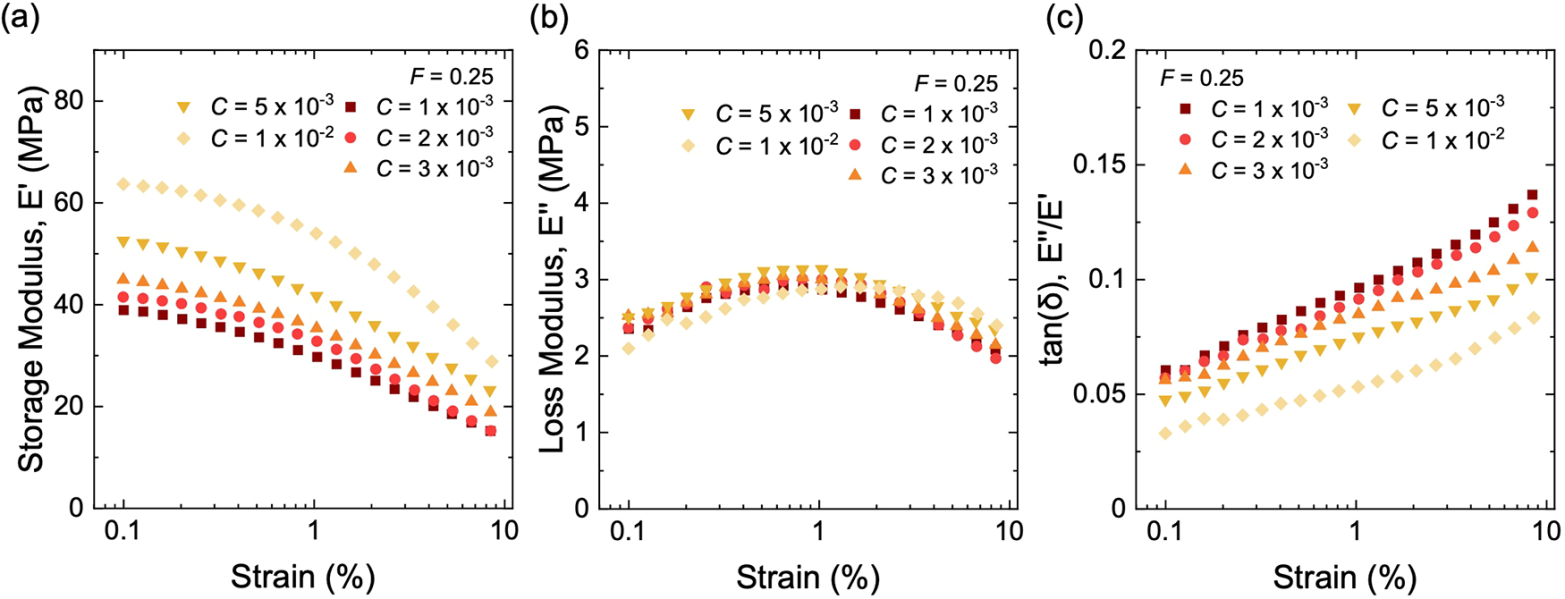
Dynamic mechanical properties for composite of long-chain natural rubber and structured silica of various initiator fractions, *C*. (a) Storage modulus, *E*′. (b) Loss modulus, *E*″. (c) tan­(δ). All samples have the same volume fraction of aggregates at *F* = 0.25.

We next discuss the experimental findings in terms of interactions of three types: rubber–rubber, rubber–filler, and filler–filler. This framework exists in the literature.^5,39–41^ The rubber–rubber interaction is analyzed using the continuum mechanics of linear viscoelasticity. In the dynamic mechanical analysis, strains are small enough and are unlikely to break covalent bonds of rubber chains, cross-links, and interlinks. Consequently, hysteresis results from noncovalent interactions between polymer chains. At small strains, the neat rubber is linearly viscoelastic: both the storage modulus *E*′ and loss modulus *E*″ are independent of the amplitude of strain (Figures 7 and 8). For natural rubber at small strains, strain-induced crystallization does not set in, the noncovalent interactions between rubber chains are weak, and the hysteresis is low; for example, tan­(δ) ∼ 0.06 for *C* = 1 × 10^–3^. The rubber is incompressible, and the linear viscoelasticity is characterized using the complex modulus *E*_m_^*^ = *E*′ + i *E*″. The complex modulus *E*_m_^*^ depends on the cross-link density, temperature, and frequency. In this paper, we fix the temperature and frequency, but vary the cross-link density.

When rubber is filled with rigid particles, at a small enough strain, the hysteresis of the composite is well represented by the continuum mechanics of linear viscoelasticity.^42,43^ That is, the complex modulus of the composite scales with the complex modulus of the rubber matrix, *E*_c_^*^ = β*E*_m_^*^, where the dimensionless real number β depends on the volume fraction and arrangement of the fillers. The storage modulus *E*′ and loss modulus *E*″ of the composite scale with those of the neat rubber in the same way, so that the hysteresis *E*″/*E*′ of the composite coincides with that of the matrix. This prediction of linear viscoelasticity is consistent with our results at small strains, e.g., 0.1–0.3% (Figures 7c and 8c). In the remainder of this article, we attribute the limit of composite and matrix having the same hysteresis to noncovalent rubber–rubber interactions alone.

In a composite, however, noncovalent interactions exist not only between rubber chains but also between rubber chains and filler particles, as well as between filler particles.^5,40,44,45^ When the strain is sufficiently small, deformation is mainly accommodated by the rubber matrix. The rubber–filler and filler–filler interactions remain nearly intact. Consequently, rubber–rubber interactions prevail and rubber–filler and filler–filler interactions contribute negligibly to hysteresis. With the increase in strain, the noncovalent rubber–filler and filler–filler interactions contribute to hysteresis. The hysteresis of the composite is no longer predicted by the continuum model of linear viscoelasticity. These remarks are consistent with our experimental data: at strains above 1%, the hysteresis of composites significantly deviates from that of the neat rubber (Figures 7c and 8c).

We attribute the difference in hysteresis between the composite and the neat rubber to the rubber–filler and filler–filler interactions. Rubber and silica are interlinked by covalent bonds, but the rubber strands between the interlinks interact with silica weakly and are mobile relative to the surface of the silica. This noncovalent rubber–filler interaction can contribute to hysteresis.^45^ Furthermore, nearby filler particles interact with each other through long-range noncovalent interactions, such as van der Waals forces.^40,44,46^ We understand such filler–filler interactions as follows. The filler particles dispersed in rubber are analogous to colloidal particles dispersed in a liquid. When the composite deforms, the filler particles move relative to one another. This relative motion of particles, in the presence of long-range noncovalent interactions and the elasticity of the rubber, leads to instability.^47^ A similar instability was observed in the experiment of Johnson, Kendall, and Roberts.^48,49^ In the JKR experiment, a sphere of a soft material is gripped by a tester. As the tester moves the sphere toward a flat surface of a rigid substrate, the force acting on the grippers is recorded as a function of the displacement of the grippers. When the surface of the sphere is near the surface of the substrate, the long-range interaction between them causes the sphere to suddenly deform and snap into contact with the substrate so that the force–displacement curve records a jump. Subsequently, when the grippers pull the sphere away from the substrate, after some small deformation, the sphere suddenly snaps and separates from the substrate, and the force–displacement curve records another jump. The recorded hysteresis in the force–displacement curve is a measure of energy loss due to long-range noncovalent interactions between the sphere and the substrate. In a composite, the nearby aggregates are separated by a gap filled with rubber. The long-range noncovalent filler–filler interactions can similarly contribute to the hysteresis of the composite. Further theoretical and computational work is required to investigate the individual contributions from the three types of noncovalent interactions to hysteresis.

## 5 Structured vs Unstructured Silica

We compare the stress–stretch curves of the composite filled with unstructured silica and the composite filled with structured silica (Figure 10a). The volume fractions of fillers of the two composites are chosen so that the two composites have a similar Young’s modulus (Figure 10b). As the stretch increases, stress increases more steeply for the composite with structured silica than the composite with unstructured silica. Consequently, a larger 100%-strain modulus is achieved for the composite with structured silica (Figure 10c).

**Figure 10:**
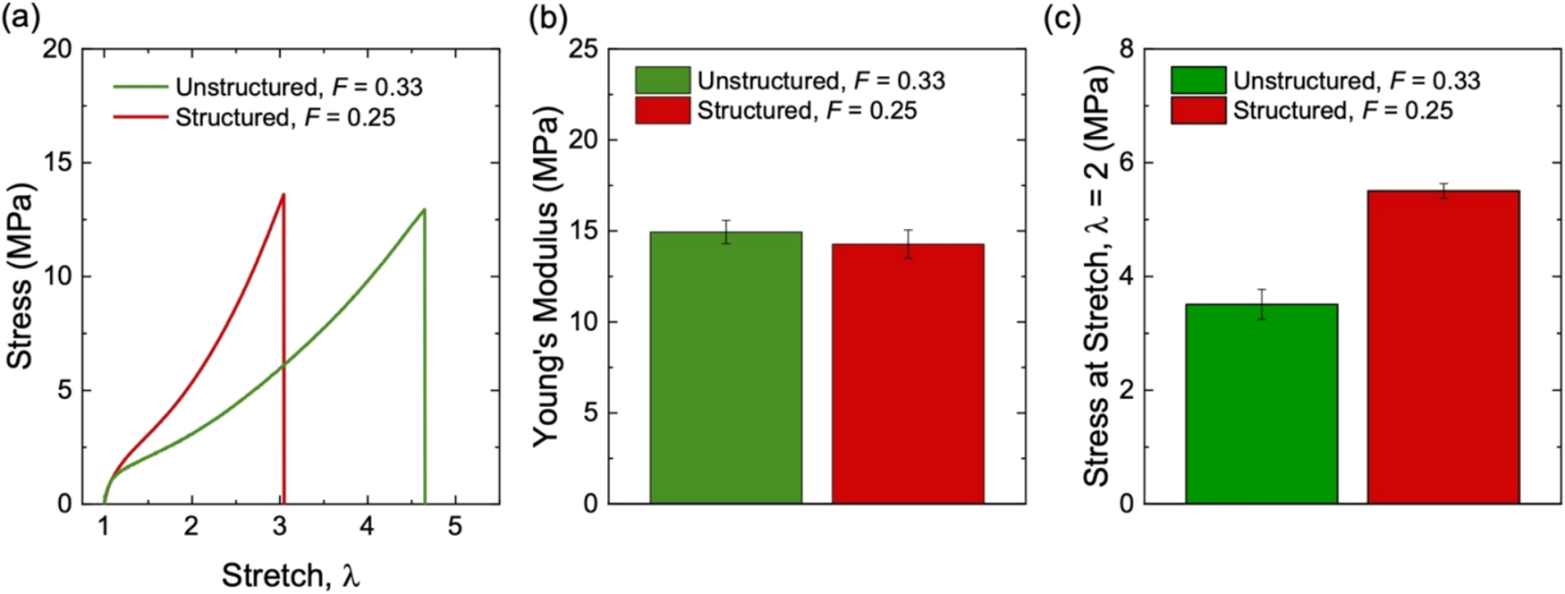
Stress–stretch behavior of long-chain natural rubbers filled with structured silica (*F* = 0.25) and unstructured silica (*F* = 0.33), respectively. (a) Stress–stretch curves. (b) Young’s modulus. (c) Stress at a stretch of λ = 2. All samples have the same molar fraction of initiator, *C* = 1 × 10^–3^.

The composite with structured silica shows a smaller reduction in *E*′ than that of the composite with unstructured silica (Figure 11a). From strain 0.1–10%, the reduction in *E*′ for the composite with unstructured silica is approximately 1.8 times that for the composite with structured silica. The loss modulus *E*″ of the composite with structured silica is lower at strains below ∼2% (Figure 11b). The peak in *E*″ occurs at a higher strain than that in the composite with unstructured silica. The hysteresis of the composite with unstructured silica is higher than that of the composite with structured silica over the entire range of strains (Figure 11c).

**Figure 11:**
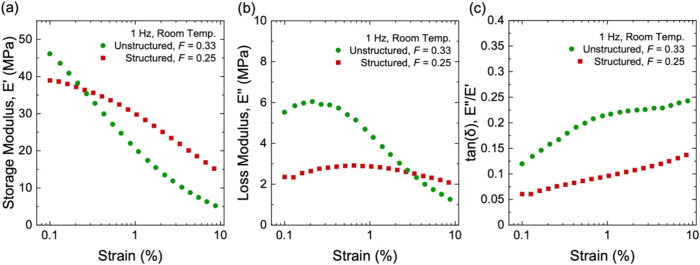
Dynamic mechanical properties of long-chain natural rubbers filled with structured silica (*F* = 0.25) and unstructured silica (*F* = 0.33). (a) Storage modulus, *E*′. (b) Loss modulus, *E*″. (c) Hysteresis, tan­(δ). All samples have the same initiator fraction of *C* = 1 × 10^–3^.

In composites filled with structured silica, a fraction of the rubber is occluded within the aggregates, amplifying the effective volume fraction of the fillers. In composites filled with unstructured silica, occluded rubber is absent, and the constrained rubber is only at gaps between nearby fillers. Achieving a comparable modulus requires a smaller volume fraction of structured silica than that of unstructured silica.

At a comparable modulus, a composite filled with structured silica achieves a lower hysteresis than a composite filled with unstructured silica (Figure 1). We interpret this finding as follows. The noncovalent rubber–rubber interactions alone predict that composites have the same hysteresis as that of neat rubber, independent of the type and volume fraction of fillers. Thus, the above difference in hysteresis of the two composites must result from the rubber–filler and filler–filler interactions. To attain a comparable modulus, a lower volume fraction of structured silica is needed than that of unstructured silica. Furthermore, the two types of silica have dissimilar feature sizes. In the unstructured silica, the average diameter of individual particles is ∼100 nm. In the structured silica, the average diameter of the primary particle is ∼12 nm. The small feature size of structured silica further reduces the volume of rubber in the gaps between the primary particles of two aggregates. As such, the volume fraction of the rubber in the gaps is higher in the composite filled with unstructured silica than in the composite filled with structured silica. The rubber in the gaps between aggregates and occluded within aggregates are likely the main contributor to the rubber–filler and filler–filler contributions. In the two composites, the volume fractions of the rubber constrained in the gaps and voids differ significantly and likely result in different hysteresis.

## 6 Structured Silica with Interlinks vs without Interlinks

To investigate the formation of interlinks between the percolated aggregate network and the rubber network, we prepared control samples filled with structured silica (Aerosil R805, Evonik) that cannot form interlinks. The structured silica used for the control samples is functionalized with octyltrimethoxysilane, making the particles hydrophobic and compatible with the liquid processing used to prepare the rubber composites. The structured silica that can interlink with rubber is functionalized with methacrylsilane. Before functionalization, the two types of silica aggregates are from the same base hydrophilic fumed silica.^27,50^ In particular, they have the same primary particle size (∼12 nm) and the same specific surface area (125–175 m^2^ g^–1^). Both composites with interlinks and without interlinks have the same volume fraction of fillers of *F* = 0.25 and initiator fraction of *C* = 1 × 10^–3^.

We compare the stress–stretch curves of composites with and without interlinks (Figure 12a). The two composites have a similar Young’s modulus (Figure 12b). As the stretch increases, the stress rises more steeply for the composite with interlinks than for the composite without interlinks. At a stretch of λ = 2, the composite with interlinks achieves a higher stress (Figure 12c).

**Figure 12:**
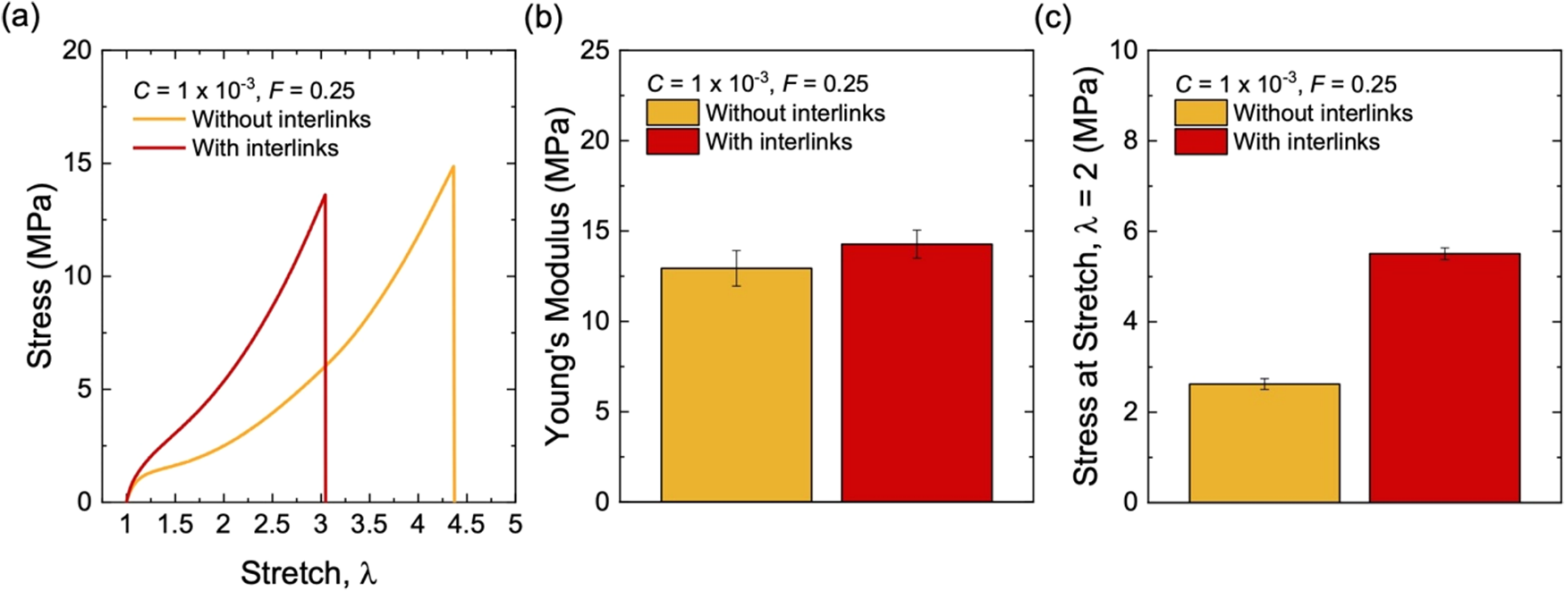
Stress–stretch behavior for composites of long-chain natural rubber and structured silica with interlinks and without interlinks. (a) Stress–stretch curves. (b) Young’s modulus. (c) Stress at a stretch of λ = 2. All samples have the same particle volume fraction of *F* = 0.25 and the same initiator fraction of *C* = 1 × 10^–3^.

Dynamic mechanical analysis further highlights the effect of interlinking on the viscoelastic response. At small strains, e.g., 0.1–0.3%, composites with interlinks and without interlinks have comparable *E*′ (Figure 13a). However, as strain increases, composites with interlinks consistently maintain a higher *E*′ value than composites without interlinks. *E*″ of both composites remains on the same order of magnitude across the measured strain (Figure 13b). As a result, composites with interlinks achieve a lower tan­(δ) with increasing strain (Figure 13c). For example, at ∼8.5% strain, composites with interlinks show *E*′ = 15.2 MPa, *E*″ = 2.09 MPa, and tan­(δ) = ∼0.14, whereas composites without interlinks show *E*′ = 9.3 MPa, *E*″ = 1.97 MPa, and tan­(δ) = ∼0.21.

**Figure 13:**
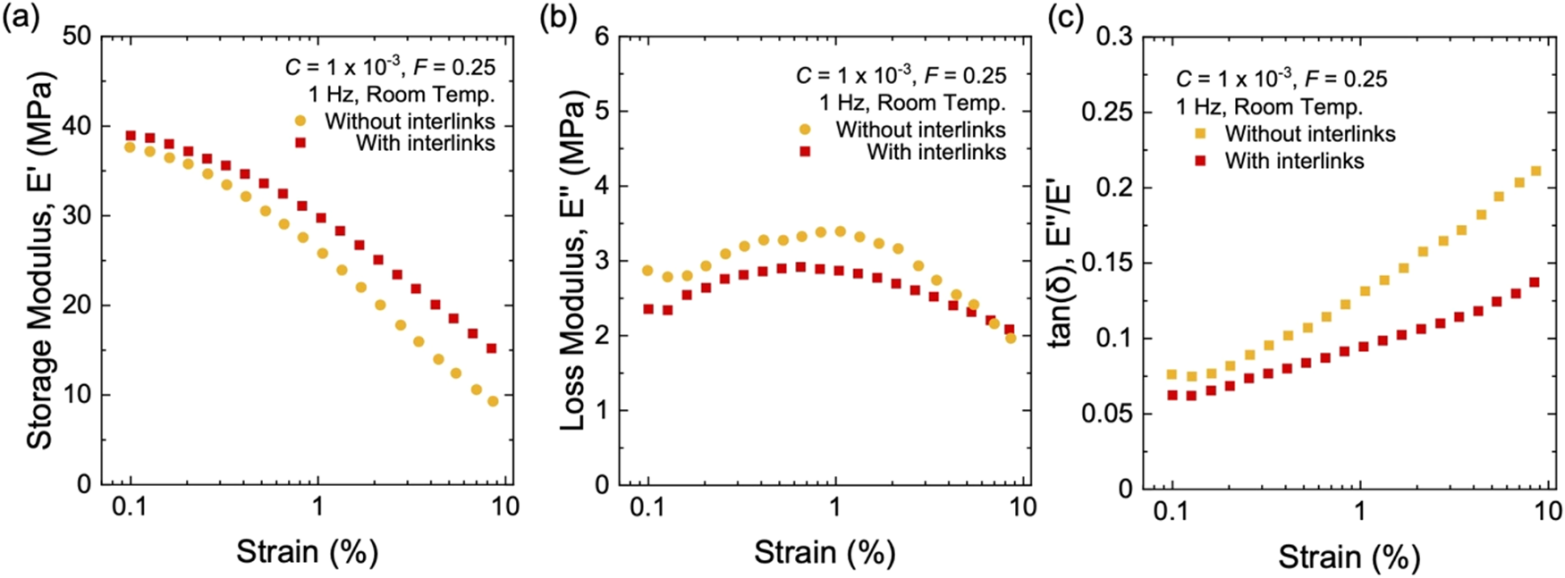
Dynamic mechanical properties for composites of long-chain natural rubber and structured silica with interlinks and without interlinks. (a) Storage modulus, *E*′. (b) Loss modulus, *E*″. (c) Hysteresis, tan­(δ). All samples have the same particle volume fraction of *F* = 0.25 and the same initiator fraction of *C* = 1 × 10^–3^.

When rubber is filled with structured silica at a sufficiently small strain, the hysteresis is independent of covalent interlinking within the composite and is well described by the continuum mechanics of linear viscoelasticity. In this limit, *E*′ and *E*″ of the composite scale with the rubber matrix, such that the hysteresis is comparable to neat rubber. However, as the strain increases, the hysteresis of the composite deviates from the prediction of linear viscoelasticity, indicating contributions from rubber–filler and filler–filler interactions. In composites without interlinks, rubber chains at the filler surface are weakly bound and slide significantly relative to fillers, while nearby aggregates can move relative to one another. Such relative motion breaks noncovalent rubber–filler interactions and promotes instability arising from filler–filler interactions, contributing to increased hysteresis. By contrast, covalent interlinks between rubber chains and structured silica constrain the motion of rubber at the filler surface and reduce the relative motion between neighboring filler aggregates. As a result, fewer noncovalent interactions are broken during deformation, leading to lower hysteresis at larger strains.

The above results demonstrate the role of covalent interlinks in reducing hysteresis at larger strains. We note that the density of interlinks cannot be independently resolved in the present system, because the radicals used for interlinking are also consumed by rubber–rubber cross-linking reactions.

## 7 Composites Prepared with Milled vs Unmilled Compounds

To study the effect of the chain length of rubber on modulus and hysteresis, we further prepare materials by milling the compound before curing. Milling cuts chains short (mastication).

### 7.1 Neat Natural Rubber

We first compare the rheology of neat natural rubber with or without milling at the same low initiator fraction of *C* = 1 × 10^–3^. For both samples, the storage modulus *E′* (Figure 14a), loss modulus *E″* (Figure 14b), and tanδ (Figure 14c) are nearly independent over the measured range of strain. The milled rubber has a much lower *E*′ and a similar *E*″ compared to the unmilled rubber. Consequently, the milled rubber has a much larger hysteresis.

**Figure 14:**
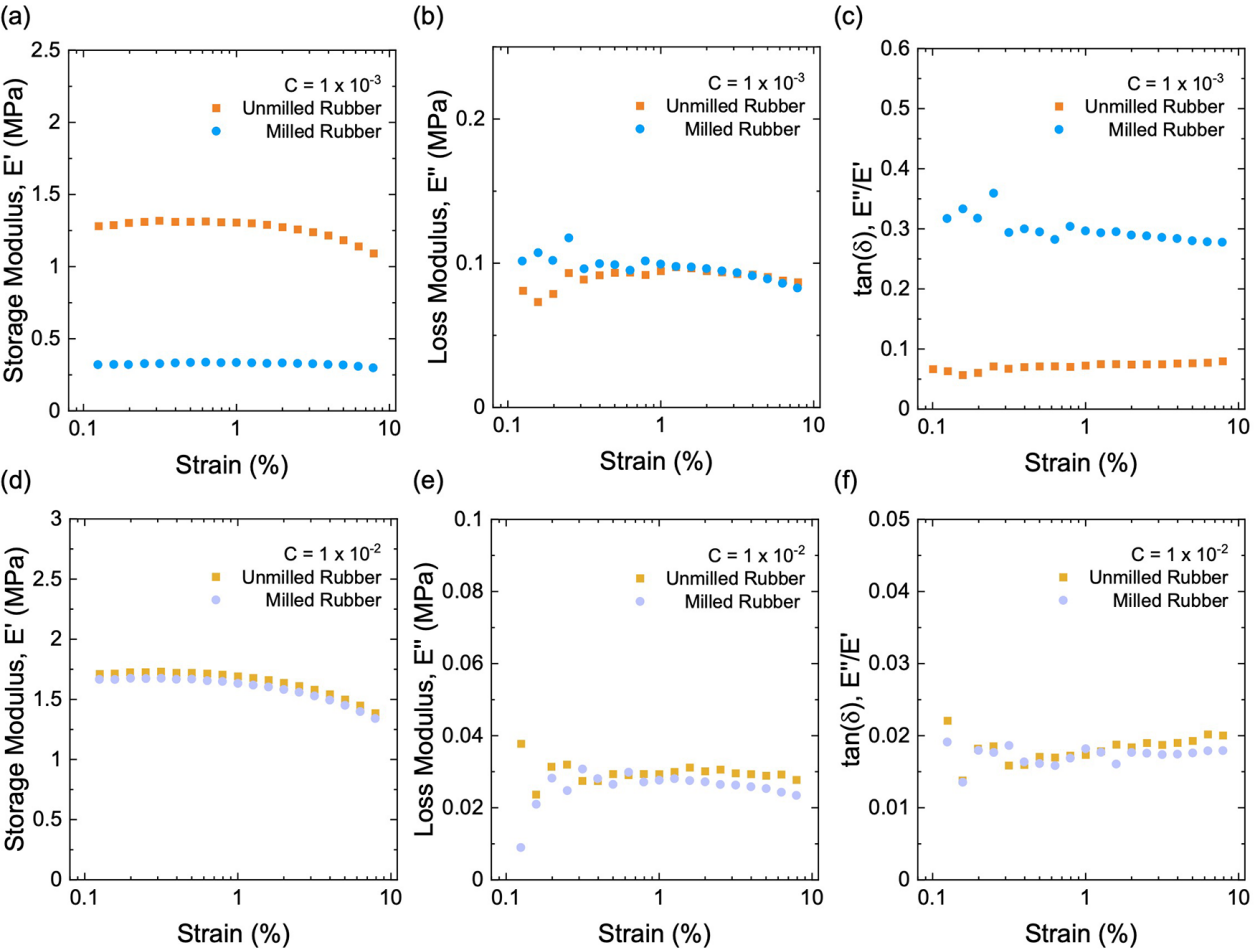
Dynamic mechanical properties for milled and unmilled neat natural rubbers. (a) Storage modulus, *E*′, (b) loss modulus, *E*″, and (c) tan­(δ) of samples at a low initiator fraction of *C* = 1 × 10^–3^. (d) Storage modulus, *E*′, (e) loss modulus, *E*″, and (f) tan­(δ) of samples at a high initiator fraction of *C* = 1 × 10^–2^.

We also compare the rheology of neat natural rubber with or without milling at a high initiator fraction of *C* = 1 × 10^–2^. For both samples, *E′* (Figure 14d), *E″* (Figure 14e), and tanδ (Figure 14f) are independent of the amplitude of strain. However, unlike the case at low *C*, milling has little effect at high *C*, with the magnitude of *E*′, *E*″, and tanδ being comparable for the milled and unmilled rubbers.

We discuss these results as follows: In the range of measurements, *E*′ and *E*″ are constant and independent of the amplitude of strain so that both the milled and unmilled neat rubbers are characterized by linear viscoelasticity. At a low initiator fraction of *C* = 1 × 10^–3^, the network is sparsely cross-linked. Milling cuts chains and increases the fraction of dangling ends after curing.^31,51^ Because dangling ends do not carry a load, the milled rubber has a lower E′ than the unmilled rubber (Figure 14a). At the same *C*, the strand length between cross-links is the same for both milled and unmilled rubbers. Since dangling ends are elastically inactive and experience little stress under small strains, they do not introduce additional energy dissipation. As a result, both samples have similar *E*″.

At a high initiator fraction of *C* = 1 × 10^–2^, the fraction of dangling ends is small for both milled and unmilled rubbers. As a result, the rheological behavior becomes dominated by the dense cross-links, so that both milled and unmilled rubbers have similar *E*′, *E*″, and hysteresis (Figure 14d–f).

### 7.2 Composites Filled with Structured Silica

We next compare milled and unmilled composites reinforced with structured silica at *C* = 1 × 10^–3^ and *F* = 0.25. The stress–stretch curves are measured for both composites (Figure 15a). The two composites exhibit a similar Young’s modulus (Figure 15b). The stress–stretch curve of the unmilled composite rises more steeply than that of the milled composite. At a stretch of λ = 2, a higher stress is achieved for the unmilled composite (Figure 15c).

**Figure 15:**
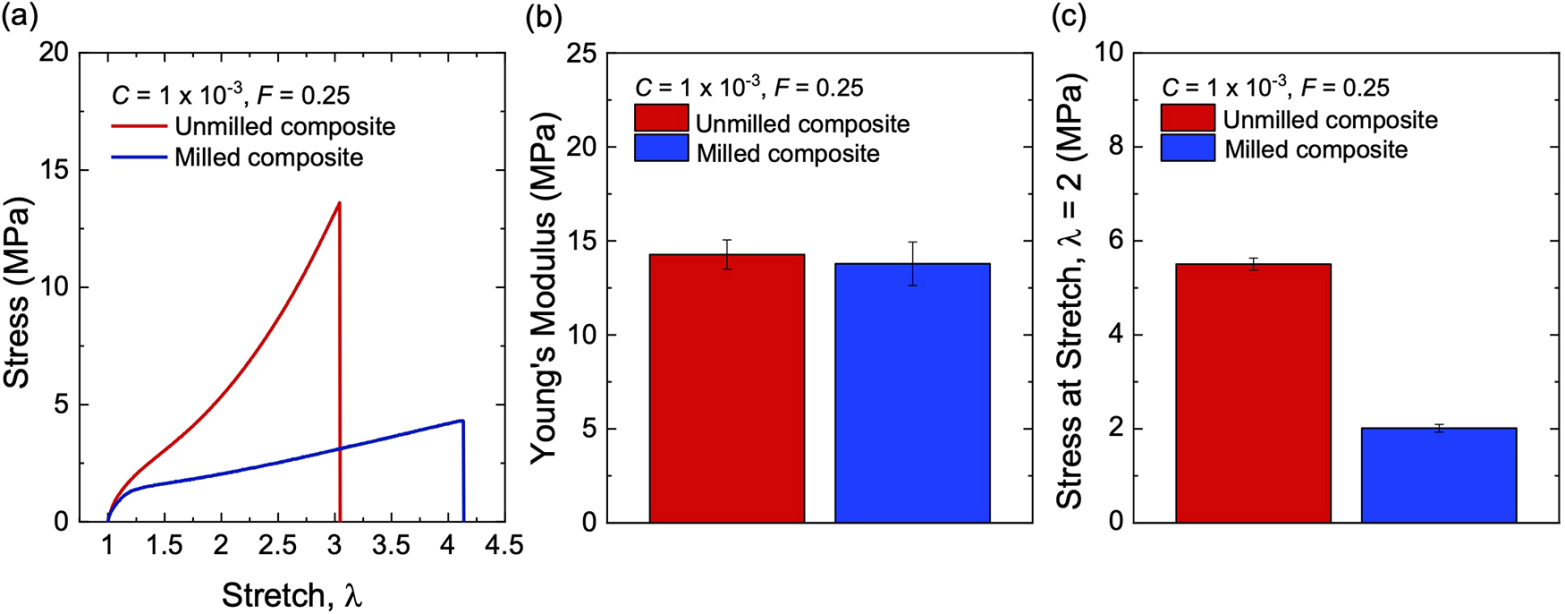
Stress–stretch behavior for milled and unmilled composites. (a) Stress–stretch curves. (b) Young’s modulus. (c) Stress at stretch λ = 2. All samples have the same particle volume fraction of *F* = 0.25, and the same initiator fraction of *C* = 1 × 10^–3^.

At the initiator fraction of *C* = 1 × 10^–3^, as the strain increases, the milled and unmilled composites show comparable reduction in the storage modulus *E*′ (Figure 16a). The milled composite shows a markedly higher loss modulus *E*″ than the unmilled composite (Figure 16b). As such, the milled composite shows a higher hysteresis than that of the unmilled composite (Figure 16c). Thus, the increased hysteresis of the milled composite is primarily associated with its larger *E*″. For example, at a strain of 1%, the hysteresis is 0.23 for the milled composite and 0.09 for the unmilled sample. As noted before, the reduction in hysteresis by a factor of more than two can potentially translate to a significant enhancement in fuel efficiency.

**Figure 16:**
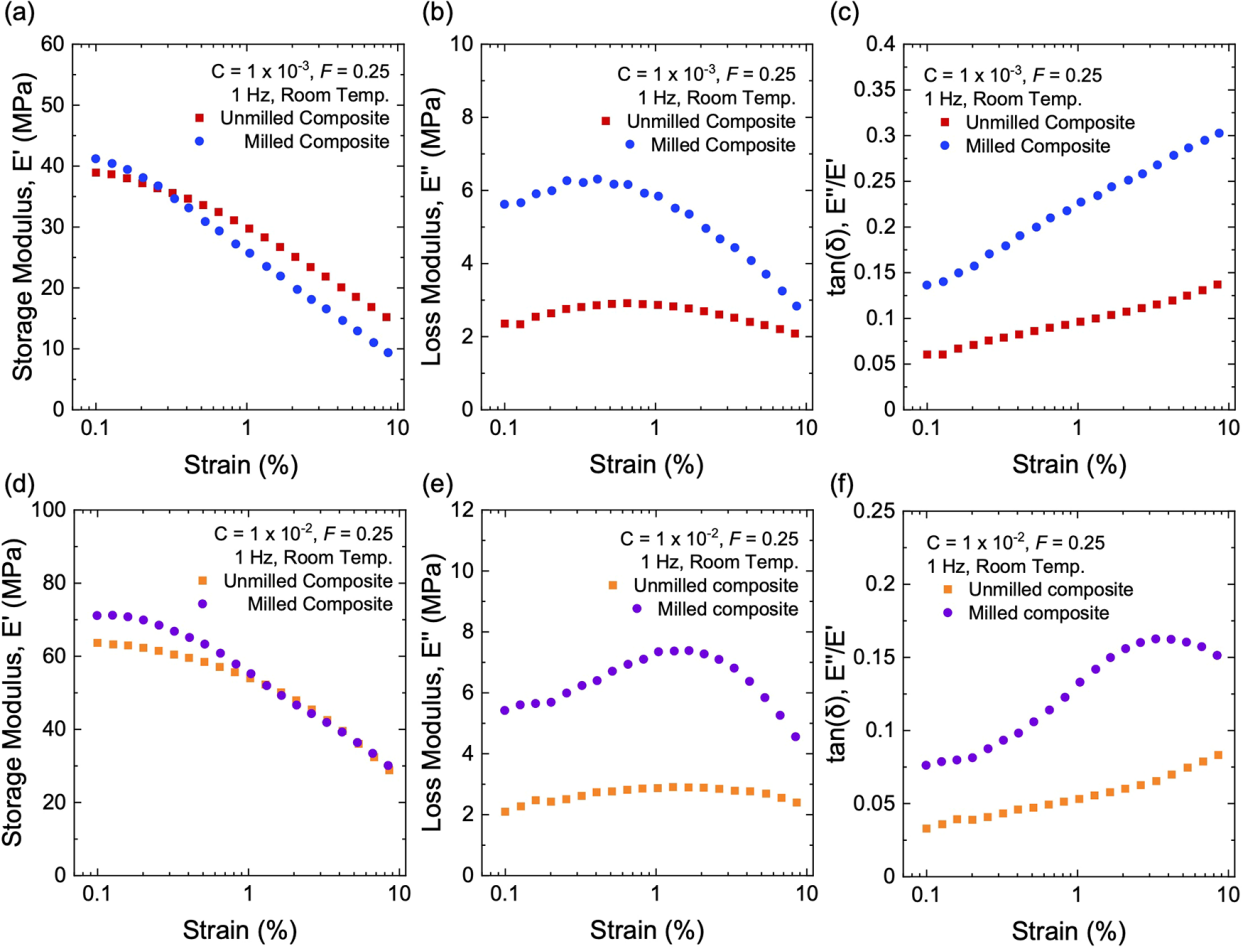
Dynamic mechanical properties for milled and unmilled composites filled with structured silica (*F* = 0.25). (a) Storage modulus, *E*′, (b) loss modulus, *E*″, and (c) tan­(δ) of samples at a low initiator fraction of *C* = 1 × 10^–3^. (d) Storage modulus, *E*′, (e) loss modulus, *E*″, and (f) tan­(δ) of samples at a high initiator fraction of *C* = 1 × 10^–2^.

We also prepare milled and unmilled composites at a high initiator fraction of *C* = 1 × 10^–2^. For either milled or unmilled composites, the sample with a higher *C* has a higher storage modulus *E*′ than that with a lower *C* (Figure 16a,d) but keeps the loss modulus *E*″ almost unchanged (Figure 16b,e). Consequently, the sample with a higher *C* has a lower hysteresis (Figure 16c,f). For both high and low initiator fractions, the unmilled composites have lower hysteresis than the milled composites.

At the same modulus, the composite with long chains achieves a lower hysteresis than the composite with short chains (Figure 1). Milling cuts chains short.^31,51^ When the cross-link density is low, the fraction of dangling ends is high, and the network is not properly formed. The effect of the rubber–rubber interaction contributes to the increase in hysteresis in the composites prepared from milled rubber. As cross-link density increases, the hysteresis of milled and unmilled neat natural rubbers becomes similar (Figure 14f). By contrast, the hysteresis of the composite prepared with an unmilled compound is appreciably lower than that of the composite prepared with milled rubber. This difference cannot be explained in terms of rubber–rubber interactions and must result from rubber–filler and filler–filler interactions. In addition, milling is a high-intensity mixing process. Besides cutting rubber chains, milling can alter the dispersion of aggregates, change the arrangement of the percolated network of aggregates, and even damage the aggregates. Further systematic studies are required to investigate the effects of milling on the microstructures and properties of the composites.

### 7.3 Recovery

We further investigate the recovery behavior of the milled and unmilled composites at *C* = 1 × 10^–3^ (Figure 17a,b). A sequence of three strain sweeps is performed: the first two sweeps are performed consecutively, followed by a third sweep after a 1 h rest period. Across all strains, the milled composite exhibits a larger drop in the storage modulus than the unmilled composite. After 1 h of rest, a large portion of the storage modulus lost with strain is recovered in the unmilled composite, whereas recovery remains limited in the milled composite. Similar trends are observed for unmilled and milled composites at high initiator fractions of *C* = 1 × 10^–2^ (Figure 17c,d). A rest period of 1 h is used because it is the shortest time examined at which full recovery is observed in the low strain region (0.1–0.3%). This time scale serves as a reference for comparing recovery kinetics between short-chain and long-chain composites.

**Figure 17:**
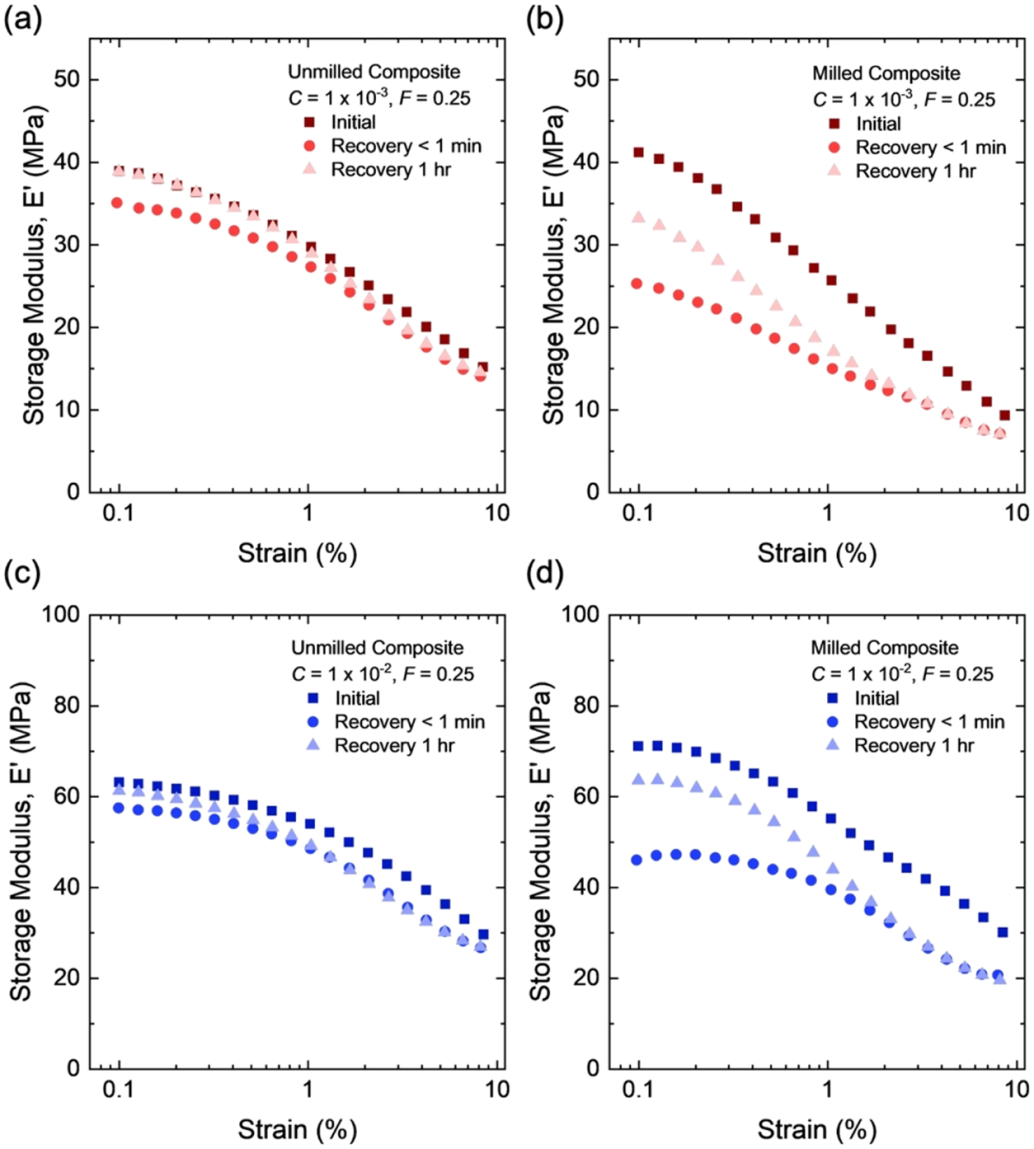
Recovery of storage modulus for composites with structured silica. (a, b) Properties of samples at a low initiator fraction of *C* = 1 × 10^–3^. (c, d) Properties of samples at a high initiator fraction of *C* = 1 × 10^–2^. The first two sweeps are performed consecutively, followed by a third sweep after a 1 h rest period. All samples have the same *F* = 0.25.

Unmilled composites show only a small change in *E*′ after the first sweep, indicating a minimal structural rearrangement of the percolated network of aggregates. These aggregates remain close to each other after deformation, enabling rapid recovery. Together, these results show that long chains and structured silica suppress irreversible particle network rearrangement and thereby reduce hysteresis.

The rubber–rubber interactions do not degrade in the dynamic mechanical analysis, as indicated by the finding that the hysteresis of neat rubber remains nearly unchanged as the amplitude of the strain increases (Figure 14). By contrast, for the composites, the hysteresis increases with the amplitude of strain. Such increases should result from changes in the rubber–filler and filler–filler interactions. Under cyclic loading, the percolated network of fillers may rearrange, breaking rubber–filler and filler–filler interactions. Rearrangement of the percolated network of fillers occurs as nearby fillers move relative to one another during cyclic loading. In the presence of noncovalent interactions and the elasticity of the rubber matrix, the relative motion of fillers gives rise to mechanical instability, which contributes to hysteresis. As fillers move, rubber strands between interlinks slide and detach from the surface of the filler, breaking rubber–filler interactions and increasing hysteresis. The lower hysteresis observed in unmilled composites suggests that long chains and structured silica can reduce the rearrangement of the percolated network of fillers during cyclic deformation.

We consider the effects of filler structure, rubber–filler interlinking, and rubber chain length on hysteresis in natural rubber composites. We next note the energy loss behavior under conditions relevant to common rubber applications. For example, in tire treads, tan­(δ) measured at 60 °C is widely used as a predictor of rolling resistance.^52–54^ In particular, it has been reported that a reduction of rolling resistance by 10% leads to a reduction in fuel consumption of 0.5–3%.^54^

Tan­(δ) of the composite with long rubber chains and structured silica is measured as a function of strain at 60 °C (Figure 18a). We compare the hysteresis of the composite (*F* = 0.25) with reported values for commercial passenger and truck tire treads (Figure 18b).^55^ The composites with long chains and structured silica have lower hysteresis than commercial tire treads under comparable small strains, indicating that the reduction in hysteresis achieved in this work reaches a level relevant to energy-efficient rubber applications.

**Figure 18:**
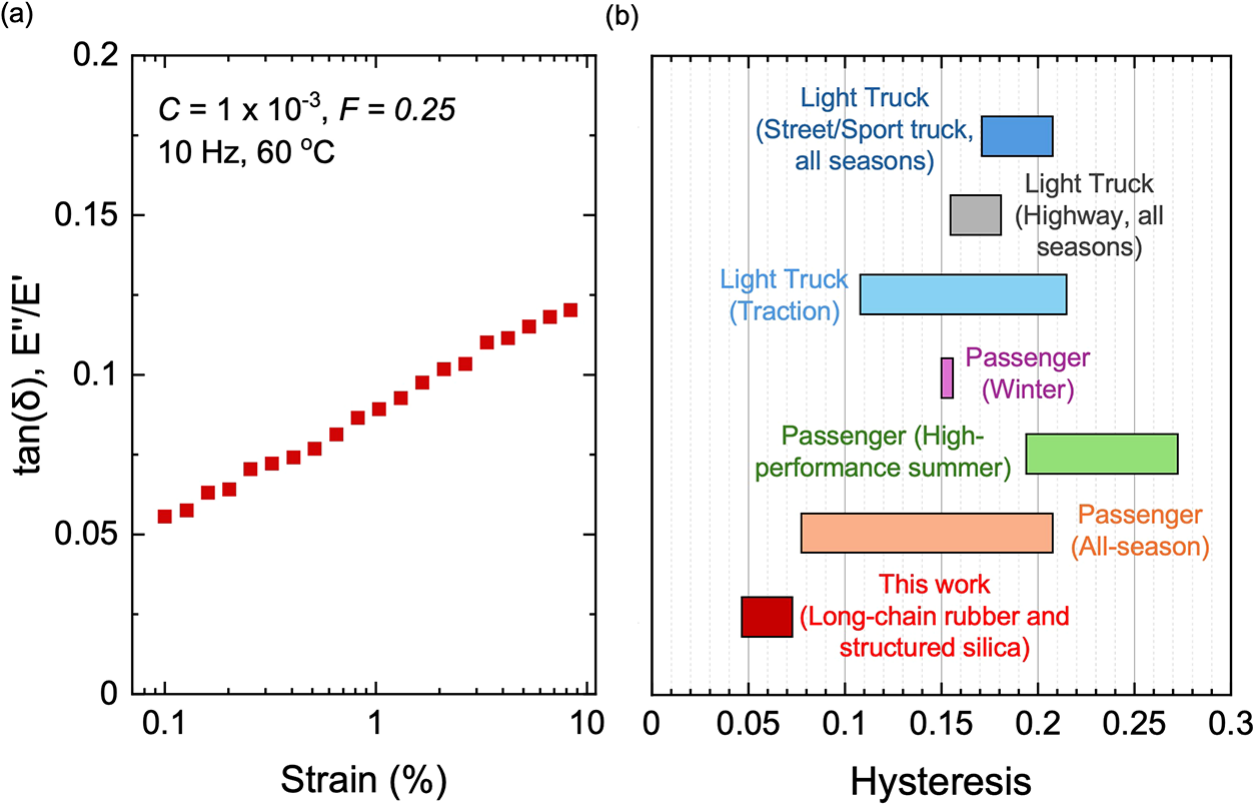
Dynamic mechanical properties at 60 °C as a predictor for rolling resistance. (a) Hysteresis tan­(δ) of the composite with long rubber chains and structured silica *C* = 1 × 10^–3^, *F* = 0.25 measured at 10 Hz. (b) Comparison of hysteresis tan­(δ), at 0.3% strain, between the composite studied in this work and commercial passenger and truck tire treads reported in the literature.^55^ Hysteresis values for the composite and tire treads were measured at 10 and 2 Hz, respectively.

## 8 Concluding Remarks

By combining long natural rubber chains with structured silica aggregates, we demonstrate that a high modulus and low hysteresis can be achieved simultaneously. The composite forms two intertwined networks: a network of cross-linked rubber chains and a percolated network of silica aggregates. By varying the molar fraction of initiator and the volume fraction of fillers, we explore mechanisms governing the modulus and hysteresis. We highlight the constraints imposed on rubber between nearby fillers and on rubber occluded within fillers. The occluded rubber increases the effective volume fraction of fillers and amplifies the modulus. Consequently, compared with composites filled with unstructured silica, composites filled with structured silica require a lower amount of silica to achieve a comparable modulus. The rubber constrained in the gaps between fillers deforms more readily than occluded rubber and is likely the main contributor to the rubber–filler and filler–filler contributions that elevate hysteresis. Because structured silica achieves modulus at low quantities and has small feature sizes, the volume of rubber constrained in these gaps is reduced, lowering hysteresis. We further show that unmilled composites exhibit lower hysteresis and better recovery after cyclic deformation than milled composites. These results provide insights for the design of filled rubber materials for applications such as tires, gaskets, and soft robotics where high stiffness and low hysteresis are desired.

## Data Availability

The authors declare that the data supporting the findings of this study are available within the article. All data are available from the corresponding author upon request.
